# Mechanical and morphological properties of additively manufactured SS316L and Ti6Al4V micro-struts as a function of build angle

**DOI:** 10.1016/j.addma.2021.102050

**Published:** 2021-10

**Authors:** Umar Hossain, Shaaz Ghouse, Kenneth Nai, Jonathan R.T. Jeffers

**Affiliations:** a Department of Mechanical Engineering, Imperial College London, South Kensington, London SW7 2AZ, UK; b Renishaw PLC, New Mills, Wotton-under-Edge, Gloucestershire GL12 8JR, UK

**Keywords:** Tensile testing, SS316L, Ti6Al4V, Micro-strut, Build angle

## Abstract

Additive manufacturing methods such as laser powder bed fusion (PBF) can produce micro-lattice structures which consist of ‘micro-struts’, which have properties that differ from the bulk metal and that can vary depending on the orientation of the strut to the build direction (the strut build angle). Characterizing these mechanical and morphological changes would help explain macro-scale lattice behavior. Individual stainless steel (SS316L) and titanium alloy (Ti6Al4V) laser PBF struts were built at 20°, 40°, 70° and 90° to the build platform, with 3 designed diameters and tested in uniaxial tension (n = 5). Micro-CT was used to quantify changes in surface roughness, eccentricity and cross-section. Average elastic modulus was 61.5 GPa and 37.5 GPa for SS316L and Ti6Al4V respectively, less than the bulk material. Yield strength was uniform over build angle for SS316L, but for Ti6Al4V varied from 40% to 98% of the bulk value from 20° to 90° build angles. All lower angle struts had worse morphology, with higher roughness and less circular cross-sections. These data should help inform micro-lattice design, especially in safety critical applications where lower mechanical performance must be compensated for.

## Highlights

• Morphology of Ti6Al4V micro-struts worse than for SS316L counterparts.
• Diameters measured externally may overestimate by 30%.
• Elastic modulus of micro-struts does not strongly depend on build angle.
• Ultimate tensile strength of Ti64AlV micro-struts strongly depend on build angle.

## Graphical Abstract

**Figure:**
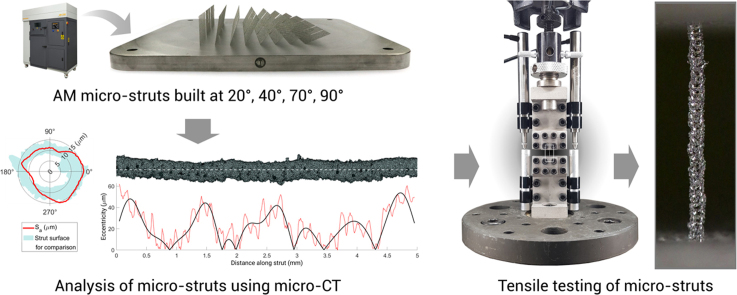

## 1 Introduction

Additive manufacturing (AM) methods such as laser-based powder bed fusion (PBF) can be used to create lattice structures made of individual beams or struts, with a diameter as low as 100 µm, also known as micro-lattice structures [1], [2], [3], [4], [5]. These structures have been manufactured in materials such as stainless steel (SS316L) and titanium alloy Ti6Al4V [2], [6], [7]. AM SS316L has high corrosion resistance and excellent weldability [8] and has been widely researched in AM literature [2], [7]. Ti6Al4V is particularly useful for its biocompatibility, and several studies have explored its use in additively manufactured bone implants [9], [10], [11]. The properties of the individual struts or ‘micro-struts’ can be significantly different to the bulk material [2], [6], [7], [12], [13], [14], and due to their small size are harder to characterize. This can impede the creation of robust FEA models of lattices and may cause unpredictable behavior in manufactured lattices.

Tensile specimens for standard material testing normally use dog-bone specimens and follow ISO 6892–1:2016 [15], but for micro-struts, it is necessary to use a modified test method due to the much smaller specimen dimensions. Established methods opt for one single strut ‘as-built’ which are gripped or fixed at each end so as to avoid slipping at the grip/strut interface [2], [7], [12], [13]. However a ‘group’ of struts in lieu of the gauge section can also be used [16], [17] and sometimes gripping ‘tabs’ are printed on the end [18], [19]. Strain has been measured in various ways. Where the crosshead extension has been used, compliance correction has to be introduced to avoid machine deflection contributing to the strain data [2], [6]. Measuring strain at the specimen itself overcomes the machine compliance issue. A clip gauge extensometer [2], [13] measures strain directly in the gauge section but can be cumbersome and may need counterbalancing due to the fragility of the specimens. Optical methods have also been used, which also measure strain along the gauge length of the specimen [7], [14]. This has also been achieved using LVDTs that are fixed between the grips, parallel to the tensile direction [12]. This method must not allow any grip slippage which can be achieved using grit paper or adhesive [6], [13].

Some mechanical properties of laser PBF micro-struts can be below that of solid AM metal. For stainless steel (SS316L), the modulus has been found to be between 37% and 74% of a bulk value of 190 GPa [2], [7], [12], [13], [20]. A reduction is also seen for the yield strength, varying between 29% and 57% of a bulk value of 494 MPa. For titanium alloy Ti6Al4V, the elastic modulus has been reported as 102 GPa and 107 GPa, 81–85% of the bulk value of 129 GPa [6], [14] (the modulus for additively manufactured bulk metal as reported by Renishaw [21]). The measurement of the strut diameter is a key step in calculating these properties and is non-trivial due to the inherent surface finish of the PBF process. Correction factors to Feret diameters have been presented [7], [14]. Another method uses the volume fraction of a lattice using a similar strut to calculate the diameter analytically [13]. Advanced imaging techniques such as SEM and micro-CT have been used to provide precise measurement of strut geometry and highlight defects such as strut ‘waviness’ and internal porosity [19], [22], [23], [24], [25], [26], [27].

The layer-wise fabrication of PBF also has an impact on the mechanical properties and morphology of single micro-struts when built vertically versus at a low angle to the build platform [16], [17], [18], [19], [25], [26], [27], [28], [29], [30], [31], [32], [33], [34], [35]. Strut build angle affects the cross section [16], [17], [18], [25], [32], ‘waviness’ or eccentricity [27], [32], [34] and roughness [28], [29], [35] of the strut. Tensile tests of struts built using laser PBF at varying angles to the build direction have been conducted [2], [30], [31], and a summary of relevant literature can be found in Table 2 in the Supplementary material. This variation with build angle can also affect the fatigue properties of lattices [36], [37]. Investigating the dependence of mechanical properties on build angle would improve the interpretation of complex lattice behavior, and potentially improve computational modeling. In the case of modeling, morphology variations affect finite element analysis models [16], [19], [33], [34], [38], [39], [40], [41], [42], in particular strut diameter, porosity and eccentricity. Introducing these defects on a statistical basis throughout the model can help improve predictions [40], but requires robust input data to define the statistical variance.

The aim of this study is to investigate how the elastic modulus and strength of micro-struts vary as a function of strut diameter and angle to the build platform, when built in SS316L and Ti6Al4V. These materials are chosen to cover a wide range of AM applications, from high specific stiffness lattices for packaging components [43] to lattices for biomechanical applications [27]. A secondary aim is to investigate the circularity of struts, their surface finish, and how different ways of defining diameter affect the calculated mechanical properties. This fundamental knowledge will improve our understanding of the mechanical properties of micro-lattices and our ability to model them using computational methods.

## 2 Materials and methods

### 2.1 Specimen manufacture

**Fig. 1:**
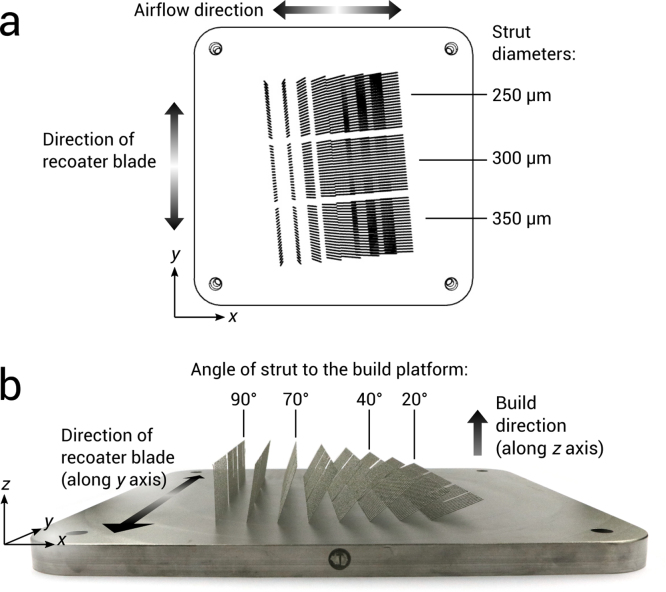
(a) Top view of CAD layout for tensile specimens, with labeled diameter groups. (b) View of the front of the build platform with Ti6Al4V specimens attached, with labeled angles used for testing.

All strut specimens were built using a Renishaw AM250 powder bed fusion (PBF) additive manufacturing system (spot size of 70 µm and wavelength of 1.07 µm). Stainless steel (SS316L) and titanium alloy (Ti6Al4V ELI, Grade 23) spherical powders were used, with a particle size range of 10–45 µm. These were supplied by Carpenter Additive Ltd. Specimens were 25.8 mm long, for an eventual 5 mm gauge length (see section 2.4) and built at a range of angles, measured as the inclination vertically from the build platform. 20°, 40°, 70°, and 90° were chosen as these cover a large range of overhang at which micro-struts of this length can be built using PBF (Fig. 1). Three designed specimen diameters were applied to the specimens, 250, 300 and 350 µm. These diameters are chosen as they are used regularly in micro-lattice structures [1], [2], [3], [4], [5].

Fig. 1 shows the resulting build platform, where 5 specimens per variable were built to make a total of 120 specimens. The strut overhang was angled in the x direction, to avoid damage by the recoater blade, which moves along the y-axis. Powder is deposited at the back of the build platform and spread to the front. A 5° anti-clockwise rotation was also applied to the struts to avoid repeated scraping of the recoater blade by the struts. Airflow was perpendicular in the x-axis.

Material Engine 1.0 was used (Betatype Ltd) to generate final build files for the machine. For each strut, the software calculates the intersection between the strut and the slice layer. At each intersection, an ellipse is calculated which reflects the angle of the strut to the build platform. Traditionally, the ellipse contour is traced by the laser and ‘hatch’ scans are used to fill in the strut cross-section, However, in this research the contour diameter is set as equal to the laser spot size (70 µm), ensuring that the center of the strut is melted (because the melt pool has a diameter larger than the laser spot size), and the strut thickness is then controlled by laser exposure time to increase the size of the melt pool, as shown in previous work [44], [45]. The laser exposure times are chosen based on the strut angle using prior data that related power/exposure time to strut diameter for different angled struts and specific laser strategy [44], to achieve the desired diameters. For SS316L, the laser power was 200 W and exposure time varied from 40 to 100 µs. For Ti6Al4V, the laser power was 50 W and exposure time varied from 50 to 600 µs. Full tables of the process parameters used for both materials (Tables 3 and 4) have been included in the Supplementary material section. The elliptical contour is traced out in points, with a spacing between points around the contours of 45 µm for all specimens. A 50 µm slice thickness was used. The method described herein is used in previous work [12].

### 2.2 Micro-CT scanning and analysis

Radiolucent strut holders were used to hold 9 struts in a 3 × 3 formation for micro CT scanning using a Bruker SkyScan 1272. Images were acquired at 1° spacing, no frame averaging and a voxel size of 3.5 µm. The source voltage and current were 100 kV and 100 µA respectively. A 0.11 mm Cu beam hardening filter was used to improve the image contrast and remove streak artifacts [46]. The volume reconstruction from X-ray images was calculated using NRecon 1.7.1.0.

#### 2.2.1 Treatment of the micro-CT voxel volume

The central 5 mm of each scan of the beam was analysed as this would be the critical gauge length section of the strut undergoing the tensile test. A threshold operation was applied to all volumes automatically, creating a binary image using Otsu’s method, as the grayscale images can be assumed to contain two classes (air or metal) and the histogram of the images is therefore bimodal [47]. This ensured consistency across all beams. Pores below 150 voxels in volume, corresponding to an equivalent pore diameter of 23.1 µm were not included and extraneous particles which were unconnected to the strut volume were removed so they did not influence any results.

#### 2.2.2 Meshing of micro-CT voxel volume

A triangular surface mesh was generated for the outer surface of the strut and inner pore surfaces using the iso2mesh meshing algorithm [48], which is a MATLAB implementation of the CGAL 3D Surface Mesh Generation library [49]. The maximum radius of the Delaunay sphere used to mesh the surfaces was 17.5 µm, lower than the average radius of the powder particles, ensuring that surface detail caused by powder particles would be captured. A tetrahedral mesh of the volume of the strut was also generated using the triangular surface mesh as an input. The maximum target volume for the tetrahedral elements was 25 voxels, equivalent to a spherical diameter of 6.35 µm.

The axis of the strut was found by fitting a line in 3 dimensions to the centroids of the resulting mesh volume elements, using a least squares method. The mesh was then realigned so that the strut axis followed the global Z-direction. For subsequent analysis, the triangular surface mesh of the strut and internal pores was intersected with perpendicular planes to find the cross-section, giving the perimeter geometry and the cross section of any pores in the plane of the cross section. 400 measurements were taken along the 5 mm gauge length of the beam, once every 12.5 µm.

**Fig. 2:**
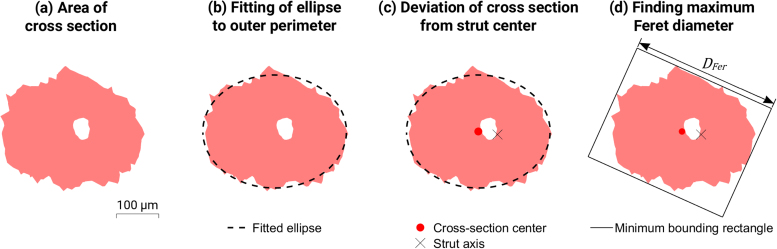
Operations on strut cross sections showing the ellipse fit and minimum bounding rectangle algorithm.

The cross-sectional area of each intersection was found (pink area of Fig. 2a). This was used to calculate an effective diameter per cross section, the results of which were averaged at the end for an average diameter (*D*_*avg*_). The minimum of these effective diameters was also recorded as the minimum diameter (*D*_*min*_). An ellipse was fitted to the outer perimeter (not including any points from pore intersections). By dividing the major axis length by the minor axis length of the resulting ellipse, the elliptical ratio was found at each measurement point along the strut. This is 1 if perfectly circular and increases as the cross-section deviates from the intended geometry (Fig. 2b).

The centroid of the ellipse was also found at each point along the strut. The distance from the centroid to the strut axis at (0,0) was found, quantifying the eccentricity of the strut along its length (Fig. 2c).

To compare to the average and minimum diameter measurements, the Feret diameter was found per cross section, which simulates the maximum diameter measurement achieved by micrometer or caliper externally (Fig. 2d). This was found by generating the minimum bounding box that contains the cross-section perimeter and using the larger of the two side measurements as the maximum Feret diameter (*D*_*Fer*_), similarly to previous work [14].

#### 2.2.3 Roughness analysis

**Fig. 3:**
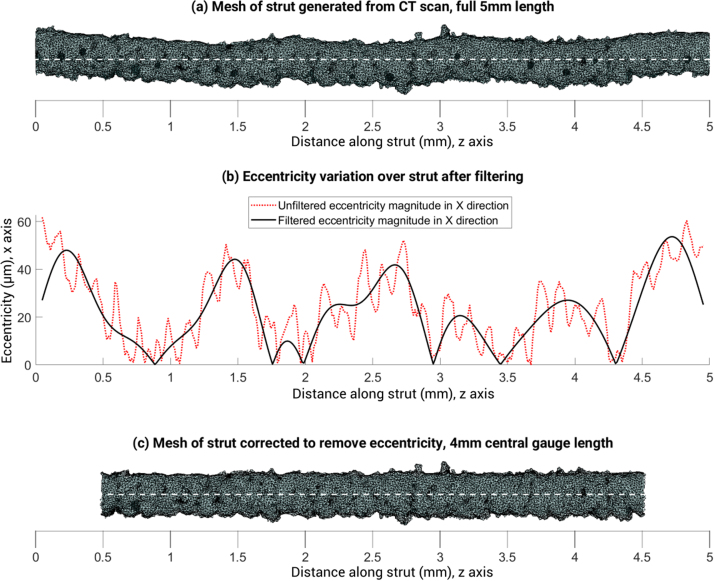
Removal of eccentricity from strut mesh before roughness analysis.

An FFT transform of the eccentricity of the strut with a lower cut off frequency of 0.5 mm over its length (Fig. 3a) was performed to remove eccentricity of the beam.

**Fig. 4:**
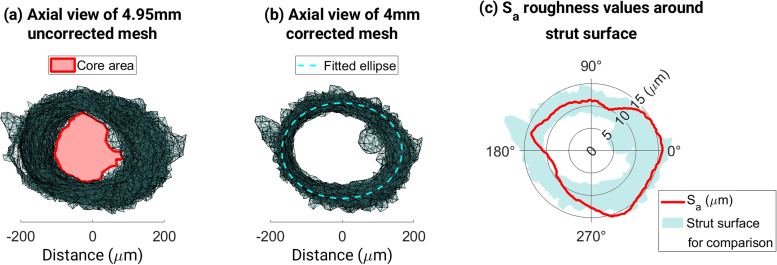
Process of calculating the core area, and the ideal elliptical prism representing the mean surface of the strut.

After filtering the FFT transform, the signal was transformed back to the correct domain and the middle 4 mm of the 5 mm gauge length was used for the roughness measurements. An ellipse was fitted to all vertices of the corrected mesh after projection into the X-Y plane (Fig. 4b) so that the mean surface could be found. The strut surface was compared to this ideal elliptical prism to find the roughness at different angular locations around the surface of the strut.

The absolute value of the deviation of each node in the mesh from this elliptical mean surface $y_{i}$ was found. This was defined by the difference between the radial height of the node from the origin and the radial distance of the elliptical surface in the same direction to the origin. Then, the deviations of the nodes within a given angular span of 10° were summed and averaged over the number of nodes n queried. This calculation (shown in Eq.) was made every 2° in an angular direction around the strut. The surface roughness $S_{a}$ could then be provided for a particular angle, as shown in Fig. 4c.

(1) $$S_{a}=\frac{1}{n}\sum_{i=1}^{n}\left|y_{i}\right|$$

The mean and deviation of the surface roughness were also found to give average results for the whole strut surface.

#### 2.2.4 Finding core area diameter

The core area diameter was defined as a solid area down the axis of the strut, which is consistent throughout the gauge length despite any eccentricity (Fig. 4a). The area of the inner core was found and a corresponding cylindrical diameter, *D*_*core*_, was calculated for comparison to other diameter measurements, *D*_*avg*_, *D*_*min*_ and *D*_*Fer*_. A summary of diameter measurements is shown in Table 1 below.

**Table 1:** Summary of diameter measurements for each strut.

Diameter measurement	*D*_*core*_	*D*_*min*_	*D*_*avg*_	*D*_*Fer*_
Description	Equivalent diameter of consistent inner core	Minimum equivalent diameter from strut mesh	Average equivalent diameter along strut	Maximum simulated micrometer diameter

### 2.3 Tensile testing

**Fig. 5:**
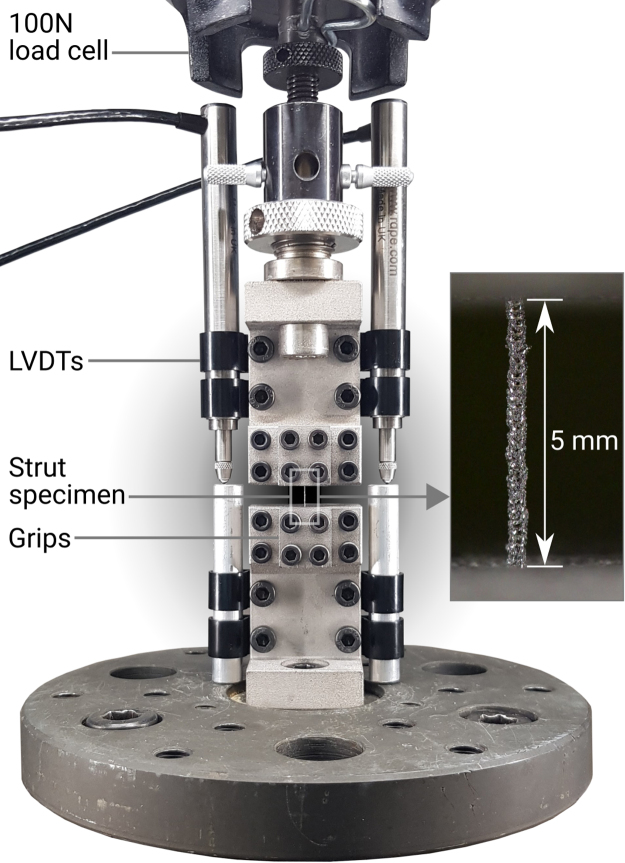
Tensile testing rig using LVDTs, showing grips and specimen gauge length.

All strut specimens were subject to uniaxial tensile tests to find their mechanical properties. Five replicates (n = 5) were tested for each combination of material (SS316L or Ti6AL4V), build angle (20°, 40°, 70° and 90°) and designed diameter (A, B and C). The total number of tensile tests performed was 120. The testing rig shown in Fig. 5 was used in a uniaxial Instron 5570 testing machine with a 100 N load cell. The strain across the gauge length was measured on opposite sides using LVDTs (RDP D6/05000 A) at 30 Hz and the average of these LVDT strain measurements were used.

Specimens were gripped between two additively manufactured surfaces, a plate with a small (~100 µm) 90° groove to align the specimen and a flat surface. The AM surface was unfinished to allow the rougher texture to aid in gripping. Video footage of each test was reviewed and any tests where slipping of the specimen was visually identified were discarded. Any tests where the failure did not occur within the gauge length were also discarded.

Quasi-static strain rates were used in testing, 1.67 × 10^−3^ s^−1^ for SS316L and 3.33 × 10^−4^ s^−1^ for Ti6Al4V specimens. These rates fall within range 2 and 3 of ISO 6892–1 A [15]. As the Ti6Al4V struts failed at much smaller strains than the SS316L, a slower strain rate was used to capture more data within the elastic range of the test.

Specimens were loaded continuously until failure, and stress-strain curves were used to calculate the elastic modulus (*E*) of each specimen, using a linear regression on points in the elastic region. This region was defined as between 5% and 30% of the ultimate tensile strength (*σ*_*UTS*_) for SS316L and between 10% and 50% for Ti6Al4V specimens. The yield strength (*σ*_*y*_) was also reported for SS316L specimens, defined as the stress at a 0.2% strain. An example stress-strain curve for a SS316L specimen can be seen in Fig. 14, in the Supplementary material.

## 3 Results

All shading in the following figures ( Fig. 6, Fig. 7, Fig. 8, Fig. 9, Fig. 10, Fig. 11, Fig. 12, Fig. 13) represent the maximum and minimum results for the five replicates tested per variable combination.

**Fig. 6:**
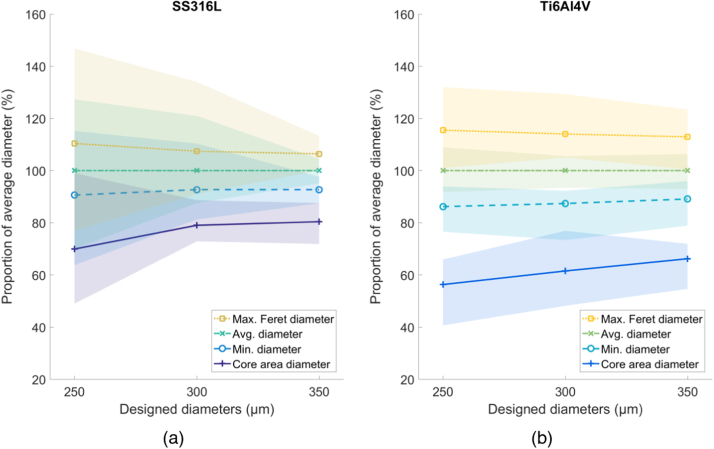
Comparison of different methods for measuring diameter for (a) SS316L and (b) Ti6Al4V struts, normalized by the average diameter. Shaded area indicates full range of results.

### 3.1 Diameter measurements

A comparison between the different methods for measuring diameter are presented in Fig. 6, where each are shown normalized by the calculated average diameter *D*_*avg*_. For both materials, *D*_*core*_ is consistently the lowest of the diameter measurements, whereas *D*_*Fer*_ is the largest. *D*_*avg*_ and *D*_*min*_ fall between the two. The variance of data tends to decrease as the designed diameter increases, suggesting less variation in the strut overall. The resulting diameters as manufactured were not always constant as a function of build angle, despite varying the laser exposure times to compensate for potential changes. The average error across all struts between the average diameter *D*_*avg*_ and the designed diameter is 11.9% (s.d. 8.3%).

### 3.2 Strut morphology

#### 3.2.1 Elliptical ratio

**Fig. 7:**
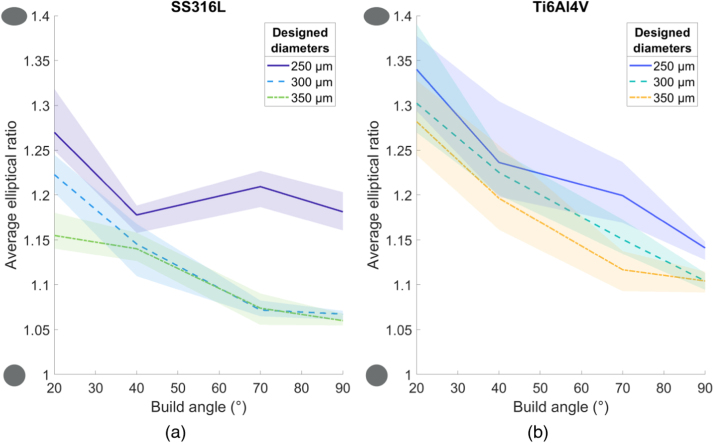
Changing elliptical ratio over build angle of the strut cross-sections for (a) SS316L and (b) Ti6Al4V struts. 1 is circular, and values above are progressively more elliptical. Shaded area indicates full range of results.

The average elliptical ratio ranges from 1.06 to 1.34 overall and gets closer to unity at higher build angles, for all struts and designed diameters. The results are shown in Fig. 7 as a function of build angle with reference cross-sections on the y-axis. For both materials, the struts are less circular at lower build angles, possibly due to the increased overhang of successive layers when the struts were being built. However, Ti6Al4V struts show slightly worse morphology than SS316L overall. The average value for the elliptical ratio across all struts is 1.17.

#### 3.2.2 Roughness

**Fig. 8:**
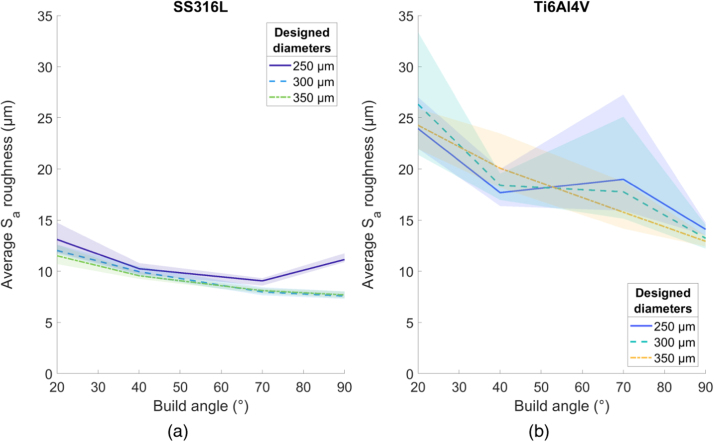
Average S_a_ roughness values over build angle, for (a) SS316L struts and (b) Ti6Al4V struts. Shaded area indicates full range of results.

Average S_a_ generally decreases at higher build angles for all parameters and materials, with a slight deviation for SS316L 250 µm struts built at 90° (Fig. 8a). Compared to Ti6Al4V struts, the variance of the SS316L data is lower. The average S_a_ roughness for Ti6Al4V struts is 18.6 µm, 1.89 times as rough as stainless-steel specimens. There is little difference in roughness between the different thickness beams for both Ti6Al4V and SS316L.

Further results showing the variation of eccentricity with build angle bear some similarity to the trends for roughness. These data can be found in the Supplementary materials.

#### 3.2.3 Porosity

The average porosity of the SS316L struts, as measured by volume fraction (pore volume divided by strut metal volume) was 0.539%. There was some reduction in porosity at low build angles (70°), though beyond this there was not a clear relationship with build angle. Across all Ti6Al4V struts, the average porosity was 0.002%, low enough to be considered negligible.

### 3.3 Tensile testing results

SS316L struts exhibited a ductile failure during testing, whereas Ti6Al4V struts failed in a brittle manner shortly after leaving the elastic regime. The mechanical properties have been compared to bulk values from the machine manufacturer datasheets, where specimens made from additively manufactured material were tested in the ‘Z′ direction, parallel to the build direction [20], [21].

#### 3.3.1 Elastic modulus

**Fig. 9:**
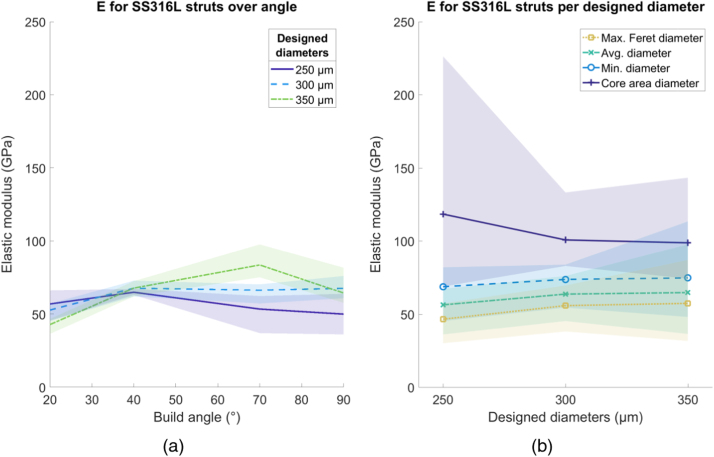
Elastic modulus (E) for SS316L struts. (a) Variation over build angle, using average diameter and (b) change in E using different diameter methods, over designed diameter. Shaded area indicates full range of results.

**Fig. 10:**
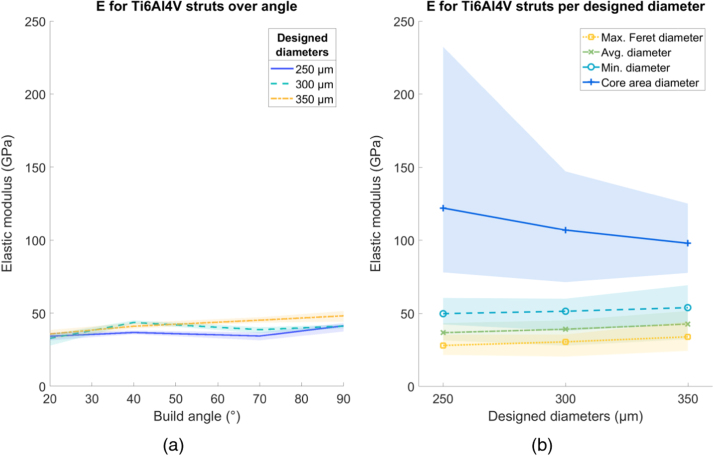
Elastic modulus (E) for Ti6Al4V struts. (a) Variation over build angle, using average diameter and (b) change in E using different diameter methods, over designed diameter. Shaded area indicates full range of results.

The average value of *E* for SS316L was 61.5 GPa, 32% of the bulk value of 190 GPa [20] (Fig. 9a). There was no clear trend for *E* changing with strut thickness or build angle. Calculating *E* using any of the diameter methods still gave a value below that of the bulk material, as shown in Fig. 9b. For Ti6Al4V struts, average *E* was 37.5 GPa, 30% of the bulk value of 126 GPa [21]. There was no clear trend for E changing with strut thickness or build angle. Calculating E using the core area diameter gave a value comparable to the bulk material, but the other diameter methods gave an E value below that of the bulk material (Fig. 10b).

#### 3.3.2 Ultimate tensile strength

**Fig. 11:**
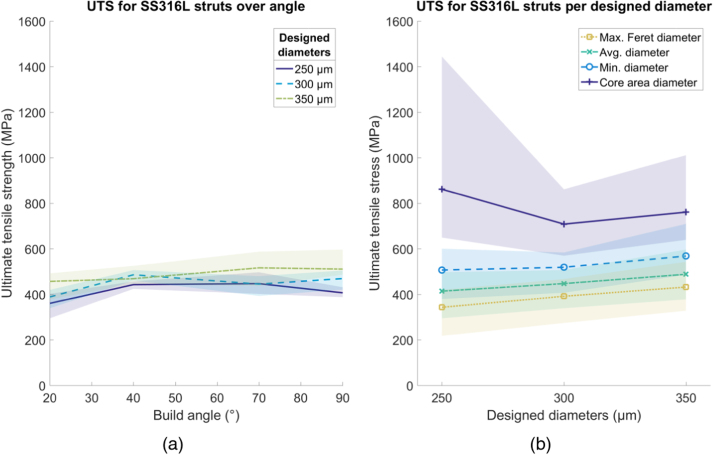
Ultimate tensile strength (σ_UTS_) for SS316L struts. (a) Variation over build angle, using average diameter and (b) change in E using different diameter methods, over designed diameter. Shaded area indicates full range of results.

For SS316L, the average *σ*_*UTS*_ across all struts, calculated using the average diameter is 450 MPa, around 72% of the bulk value of 624 MPa [20], as seen in Fig. 11a. There was no clear change in *σ*_*UTS*_ with either build angle or strut thickness. Using the core area diameter gave a *σ*_*UTS*_ value greater than *σ*_*UTS*_ of the bulk material, using the other diameter methods gave a *σ*_*UTS*_ less than the *σ*_*UTS*_ of the bulk material (Fig. 11b).

**Fig. 12:**
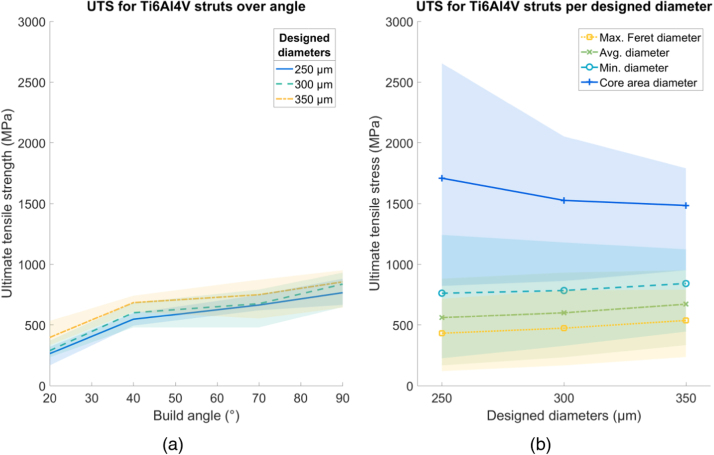
Ultimate tensile strength (σ_UTS_) for Ti6Al4V struts. (a) Variation over build angle, using average diameter and (b) change in E using different diameter methods, over designed diameter. Shaded area indicates full range of results.

For Ti6Al4V the average value of *σ*_*UTS*_ across all struts is 610 MPa, around 56% of the bulk value of 1085 MPa [21] as shown in Fig. 12a. There is a trend for increased *σ*_*UTS*_ as a function of build angle, with the strongest specimens (*σ*_*UTS*_ = 750 MPa) built at 90° to the build plate and the weakest specimens (*σ*_*UTS*_ = 290 MPa) built at 20° to the build plate. There is no clear influence of strut diameter on *σ*_*UTS*_. Using the core area diameter gave a *σ*_*UTS*_ value greater than the *σ*_*UTS*_ of the bulk material. Using the other diameters gave a *σ*_*UTS*_ less than the *σ*_*UTS*_ of the bulk material (Fig. 12b).

**Fig. 13:**
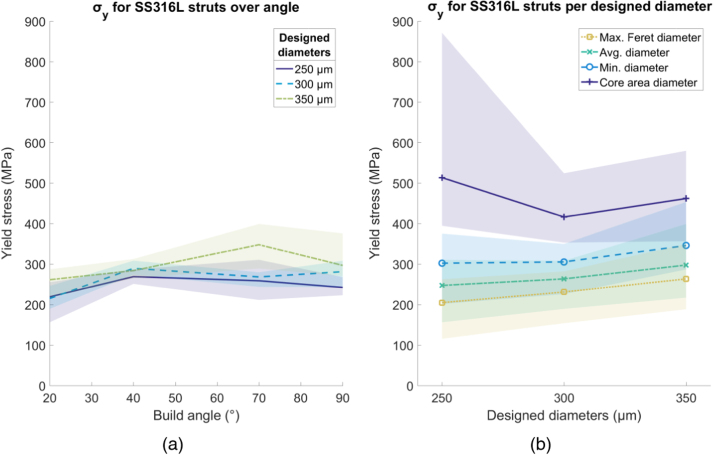
Yield stress (σ_y_) for SS316L struts. (a) Variation over build angle, using average diameter and (b) change in yield stress using different diameter methods, over designed diameter. Shaded area indicates full range of results.

#### 3.3.3 Yield strength

The yield strength *σ*_*y*_, calculated using the average diameter, is shown as a function of build angle in Fig. 13a for SS316L struts. The average *σ*_*y*_ found across all struts was 269 MPa, proportionally 55% of the bulk value of 494 MPa [20]. There is no clear relationship between *σ*_*y*_ and strut diameter or build angle. Calculation of *σ*_*y*_ using the core diameter gives values higher than exist for the bulk material, while the other diameter methods underestimate *σ*_*y*_ compared to the bulk material (Fig. 13b).

## 4 Discussion

The most important findings of this work are that the elastic modulus of both the AM SS316L and Ti6Al4V material, and the strength of the SS316L material, do not change as a function of build angle or strut diameter, when calculated using the average diameter. The strength of the Ti6Al4V material was not affected by diameter but was affected by build angle, more than doubling for a strut built at 90° compared to one built at 20°. For both materials, as the build angle increased, the surface roughness decreased, and the circularity increased. This relationship was more pronounced for the Ti6Al4V material. The Ti6Al4V struts were less ductile than the SS316L struts and the increased surface roughness and associated stress concentration points for Ti6Al4V struts may explain the increased sensitivity of strength to build angle. This study also demonstrated how the definition of strut diameter affects all the properties and may partly explain the reduced stiffness of the struts compared to the parent material. These findings enable better understanding of the mechanical performance of lattice structures. Furthermore, the mechanical data could be used to develop an accurate material model for lattice simulations and the morphology data could be used to model structural imperfections, which could reasonably be expected in lattice structures.

For SS316L struts, the elastic modulus was calculated as 61.5 GPa, 32% of the bulk value, lower than previously reported 71 GPa, 84 GPa and 140 GPa (37%, 44% and 74% of the bulk value respectively) [2], [7], [12], [13]. Previous work has investigated the impact of diameter on the microstructure and mechanical properties of vertically built SS316L struts, testing diameters from 0.25 mm to 5 mm [50]. Struts with a sub-millimeter diameter had a reduced microhardness, and the yield strength also decreased with diameter. The elastic modulus was not reported, however the effects of changing microstructure in SS316L micro-struts may also explain the variation in reported stiffness. Another work showed a decreasing trend in modulus, as specimen width is decreased to 0.4 mm [51]. The change in apparent stiffness may be related to the inherent strut shape error which becomes magnified for struts of smaller diameter and relative long length as used in our study. The yield stress *σ*_*y*_ found for SS316L struts of 269 MPa is in the range of values previously reported, between 144 and 380 MPa [2], [50]. The ultimate tensile strength found as 450 MPa is also within the range of values previously reported for SS316L struts, 347.9 MPa and 575.3 MPa [12], [51]. Table 2 in the Supplementary material section includes a comparison of the previous SS316L results discussed.

Less data exists for Ti6Al4V struts, although the elastic modulus *E* has been calculated before as 102 GPa and 107 GPa, 81–85% of the bulk value [6], [14], which is higher than our value, calculated as 37.5 GPa, 30% of the bulk value. The average value of 818 MPa for the ultimate tensile stress (*σ*_*UTS*_) of Ti6Al4V struts built at 90° can be compared with a value of yield stress found previously of 997 MPa [14], as the struts in this study failed immediately after yielding. Differences may be related to how the cross section is measured. Previous work has used a correlation between maximum Feret diameter and the average diameter as observed in sectioned struts by SEM, which may underestimate the diameter and thus overestimate stiffness [14]. It is also possible that there is a difference in Ti6Al4V microstructure, as the prior work used a laser power of 120 W, whereas the struts in this work were built at 50 W. Ti6Al4V lattices have been shown to be slightly stiffer in compression when built at 200 W versus 50 W [44]. Microhardness of laser PBF Ti6Al4V has also shown to increase with increased energy input [52], [53]. Lastly, the lack of heat treatment for the Ti6Al4V struts may also contribute to poorer mechanical performance. Heat treatment could reduce residual stress, produce a more uniform grain structure and significantly improve the mechanical properties [54], [55]. This is especially true as the struts were built at a relatively large layer thickness. The two previous studies of Ti6Al4V struts discussed are included in Table 2 in the Supplementary material section.

Methods for quantifying differences in diameter measurement have also been reported [26], A geometrically equivalent cylinder, and a numerical equivalent cylinder have been defined, the latter found by conducting FEA on voxelized models of struts to calculate an effective stiffness. These measures are both smaller than the nominal designed diameter, consistent with our results, and also varied over build angle. The comparison between the Feret diameter and the true diameter has also been explored before, showing an overestimation [14]. The average diameter *D*_*avg*_ was used to calculate *E*, *σ*_*y*_, and *σ*_*UTS*_. The true effective diameter of these varying struts probably falls somewhere between *D*_*avg*_ and *D*_*min*_. When calculating *E*, variation in the strut makes it less stiff than a completely straight and consistent beam with the same average diameter. so *D*_*avg*_ may be an overly optimistic measure. For *σ*_*y*_, yielding may occur at a few different sites along the beam that are thinner than *D*_*avg*_ and closer to *D*_*min*_, the very minimum diameter along the beam. Figs. 9b-13b show how a smaller diameter measurement increase the calculated results.

The elliptical ratio and how it changes over build angle has also been explored [16], [18], [25], [26], [32]. It has been shown to vary from 1 to 2 when building Ti6Al4V struts using EBM manufacturing [26], and as varying between 1.09 and 1.63 for Ti6Al4V struts built using powder bed fusion in the X, Y and Z direction [32] (when calculated using the method in our study). The roughness of Ti6Al4V laser PBF struts have also been shown to vary over build angle [25], [28], [35]. Weißman et. al showed that struts built at 90° and 45° had significantly different R_a_ values (p < 0.001), with higher roughness values at 45° [25]. Alghamdi et al. showed a similar trend [35], which agree with the findings of our study.

This study investigated the effect of build angle and strut diameter on material properties, but other variables such as wiper blade direction, air flow direction, layer thickness, support of the strut in the build (how likely it is to move in the building process) and availability of heat dissipation all may play a part in changing the morphological properties of the strut. A limitation of this work is that we compared our data against bulk material properties of specimens that were built on a different Renishaw AM250 machine. There may have been some small differences between the machines (same model), but we only used these data for comparison purposes and not to generate our own results. Another limitation is that specimens were printed as one long 25.8 mm strut. Struts as part of a lattice normally have nodal connections that support the strut in the build process and may have different morphological properties as a result. However, for accurate measurement of mechanical properties enough length was needed to grip the specimen. A gauge length of 5 mm was chosen for higher micro-CT accuracy, and longer gauge lengths have not been shown to change the measured mechanical properties [6].

A specific build strategy was used in the manufacturing of these struts, as described in Section 2.1, which is ‘points’ based. This differs from the more traditional ‘contour and hatch’ strategy used for other (usually larger) AM components. Therefore, the results only apply to struts built in the same way. Microstructural analysis and fractography were not performed. These would also have added further context to the failure modes of the specimens.

LVDTs were used in this study, assuming that the grip of the specimen is adequate to avoid slippage. Only specimens with no visible slippage were included in the study after reviewing video footage of the test and assessing the stress strain curves for any evidence of slipping. Digital image correlation (DIC) methods may prove a way to investigate strain distribution along a strut [56], though DIC data requires time consuming post-processing.

A final limitation is that the calculation method for the roughness value S_a_ is closer to an area roughness value and is not directly comparable to an R_a_ value that is captured using more sophisticated and dedicated hardware. This value can still be used however to compare between struts within this study. As the value comes from interrogating the mesh of each strut, the measure is still subject to any deviation of the mesh from the true metal surface, although the element size was picked to capture sub-particle details. Similar micro-CT and mesh based roughness measurements have been used in the literature before [28], [57].

The outcome of this work has presented a detailed study on the effects of build angle and strut morphology on the mechanical properties of individual struts. For steel struts, the material strength is uniform across build angles and diameters, but for titanium the strength is highly dependent on build angle, being stronger at more vertical build directions. The lower angle struts have worse morphology, being rougher and less circular in cross-section, which may explain weaker titanium struts at lower build angle where imperfections in surface may contribute to a lower tensile strength. These data should help inform the design and manufacture of AM lattices by allowing struts at different angles to be assigned stiffness and strength properties based on these experimental measurements. For lattices that may be used in safety critical applications like implantable medical devices [58], extra care must be taken to compensate for mechanical properties that may be below the bulk value, and change depending on orientation of the lattice to the build direction.

## CRediT authorship contribution statement

**Umar Hossain:** Conceptualization, Methodology, Software, Investigation, Data curation, Writing - original draft. **Shaaz Ghouse:** Methodology, Writing - review & editing. **Kenneth Nai:** Resources, Funding acquisition. **Jonathan Jeffers:** Writing - review & editing, Supervision, Project administration, Funding acquisition.

## Declaration of Competing Interest

The authors declare the following financial interests/personal relationships which may be considered as potential competing interests, Kenneth Nai is an employee of Renishaw plc, and we receive research funding from Renishaw plc.
