# Supplementary material for: Mechanical and morphological properties of additively manufactured SS316L and Ti6Al4V micro-struts as a function of build angle
Source: Addit Manuf. 2021 Oct;46:None. doi: 10.1016/j.addma.2021.102050 (PMC8448581; doi:10.1016/j.addma.2021.102050)
Supplement: Supplementary file 1 — Supplementary material. [file mmc1.docx]

**Supplementary material**

#### Previous work

Table 2: Comparison of mechanical properties found for micro-struts built using laser PBF in SS316L and Ti6Al4V

| Material | $E$ (GPa) | $\frac{E}{E_{s}}$ | $\sigma_{y}$ (MPa) | $\frac{\sigma_{y}}{{(\sigma_{y})}_{s}}$ | $\sigma_{UTS}$ (MPa) | $\frac{\sigma_{UTS}}{{(\sigma_{UTS})}_{s}}$ | Strain measurement | Author |
| --- | --- | --- | --- | --- | --- | --- | --- | --- |
| SS316L | 140 | 0.74 | 144 | 0.29 | - | - | Clip-gauge | [2] |
| SS316L | 97 | 0.51 | 250 | 0.51 | 450 | 0.72 | Clip-gauge | [13] |
| SS316L | 71 | 0.37 | 280 | 0.57 | - | - | Optical method | [7] |
| SS316L | 81.3 | 0.43 | 263.3 | 0.53 | 575.3 | 0.92 | LVDTs | [12] |
| SS316L | - | - | ~340 | 0.69 | ~480 | 0.77 | Crosshead disp. | [30] |
| SS316L | - | - | ~380 | 0.77 | ~500 | 0.80 | Crosshead disp. | [50] |
| SS316L | 71.4 | 0.38 | 317.9 | 0.64 | 347.9 | 0.56 | Digital image corr. | [51] |
| Ti6Al4V | 102 | 0.81 | - | - | - | - | Compliance corr. | [6] |
| Ti6Al4V | 107 | 0.85 | 997 | 1.01 | - | - | Optical method | [14] |

#### Process parameters

Table 3: Laser power and exposure times for SS316L specimens

|  |  | A – 250 µm | B – 300 µm | C – 350 µm |
| --- | --- | --- | --- | --- |
| Angle | Power (W) | Exposure (µs) | Exposure (µs) | Exposure (µs) |
| 20° | 200 | 70 | 90 | 100 |
| 40° | 200 | 60 | 70 | 100 |
| 70° | 200 | 50 | 60 | 80 |
| 90° | 200 | 40 | 60 | 80 |

Table 4: Laser power and exposure times for Ti6Al4V specimens

|  |  | A – 250 µm | B – 300 µm | C – 350 µm |
| --- | --- | --- | --- | --- |
| Angle | Power (W) | Exposure (µs) | Exposure (µs) | Exposure (µs) |
| 20° | 50 | 200 | 300 | 400 |
| 40° | 50 | 300 | 400 | 600 |
| 70° | 50 | 300 | 400 | 600 |
| 90° | 50 | 300 | 600 | 600 |

#### Example SS316L stress-strain curve


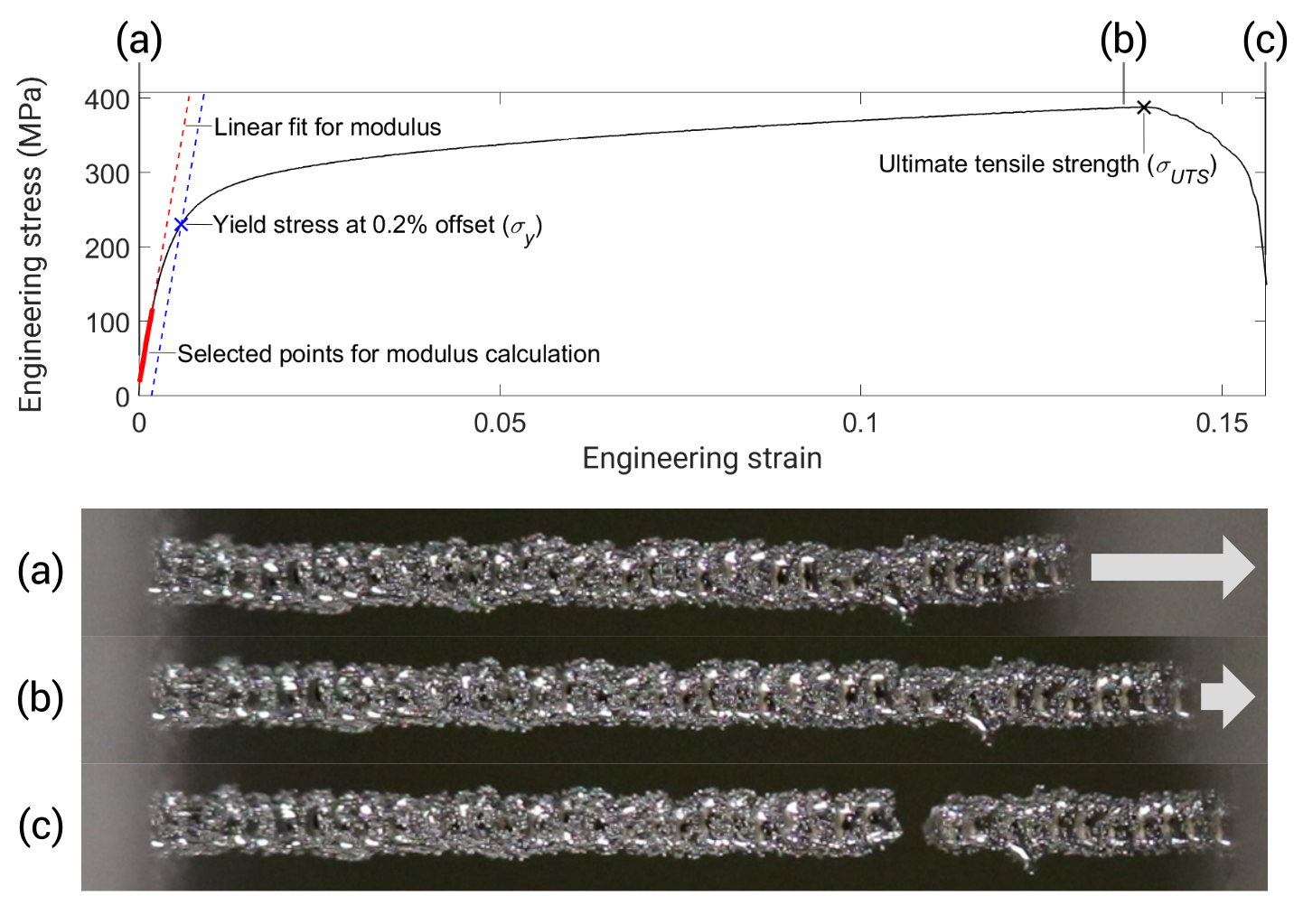


Figure 14: Example stress-strain curve for a stainless steel specimen, built at 20° to the build platform. (a) Specimen shown at the beginning of the test, (b) just before necking, and (c) after fracture.

#### Eccentricity

Eccentricity is shown as a function of build angle in Figure 15. The average eccentricity across all struts was 22.4 µm. There is a general trend to smaller amounts of eccentricity at high build angles for both materials. For SS316L struts the average eccentricity decreased from 26.1 µm at 20° to 12.0 µm at 90°, more than halving the value. A similar trend is seen for Ti6Al4V specimens, with the average eccentricity almost halving from 36.1 µm to 18.7 µm over the range of measurement. The amount of variation for Ti6Al4V specimens is larger than for SS316L and there is not a clear difference between struts with different designed diameters.

| 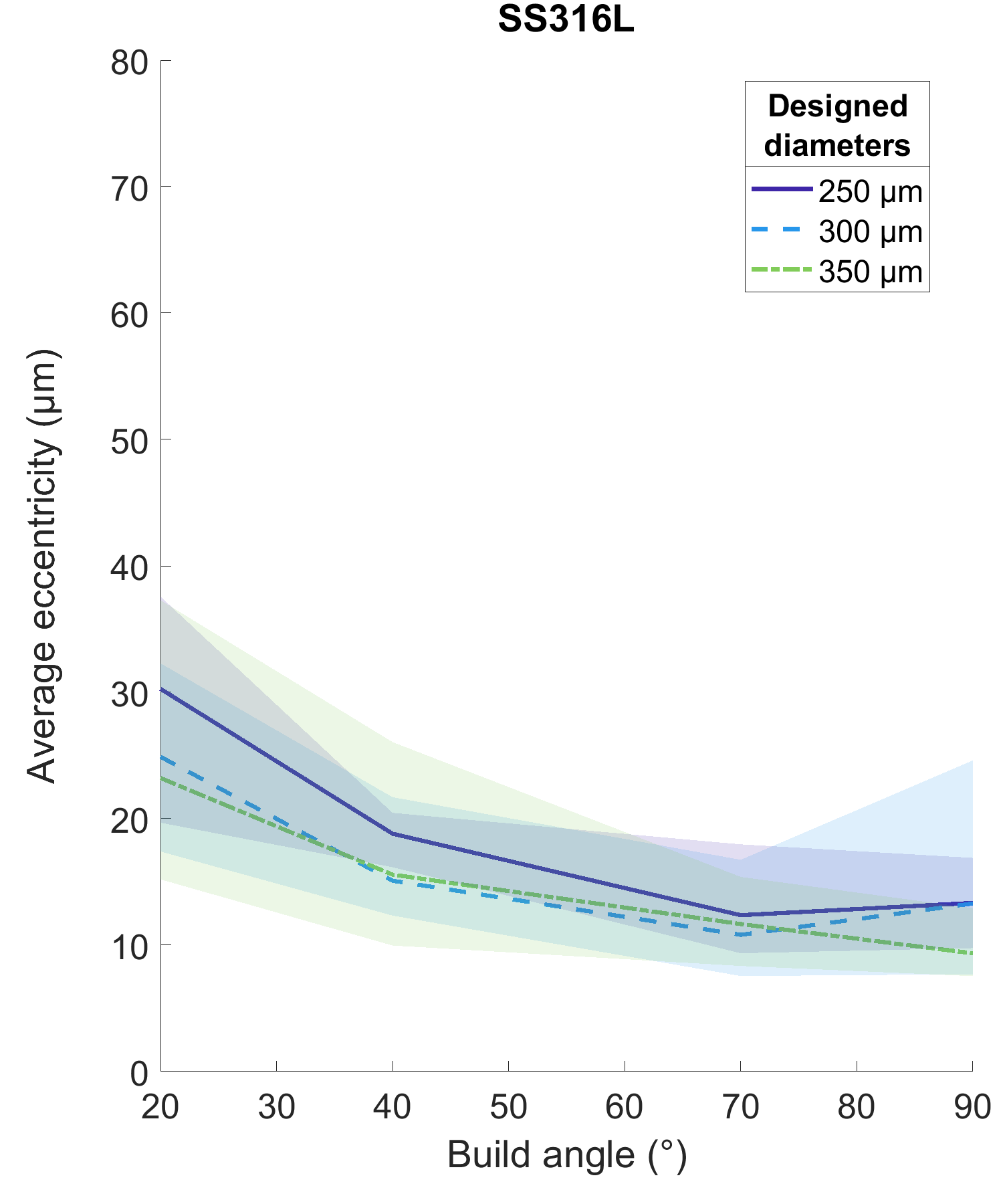 (a) | 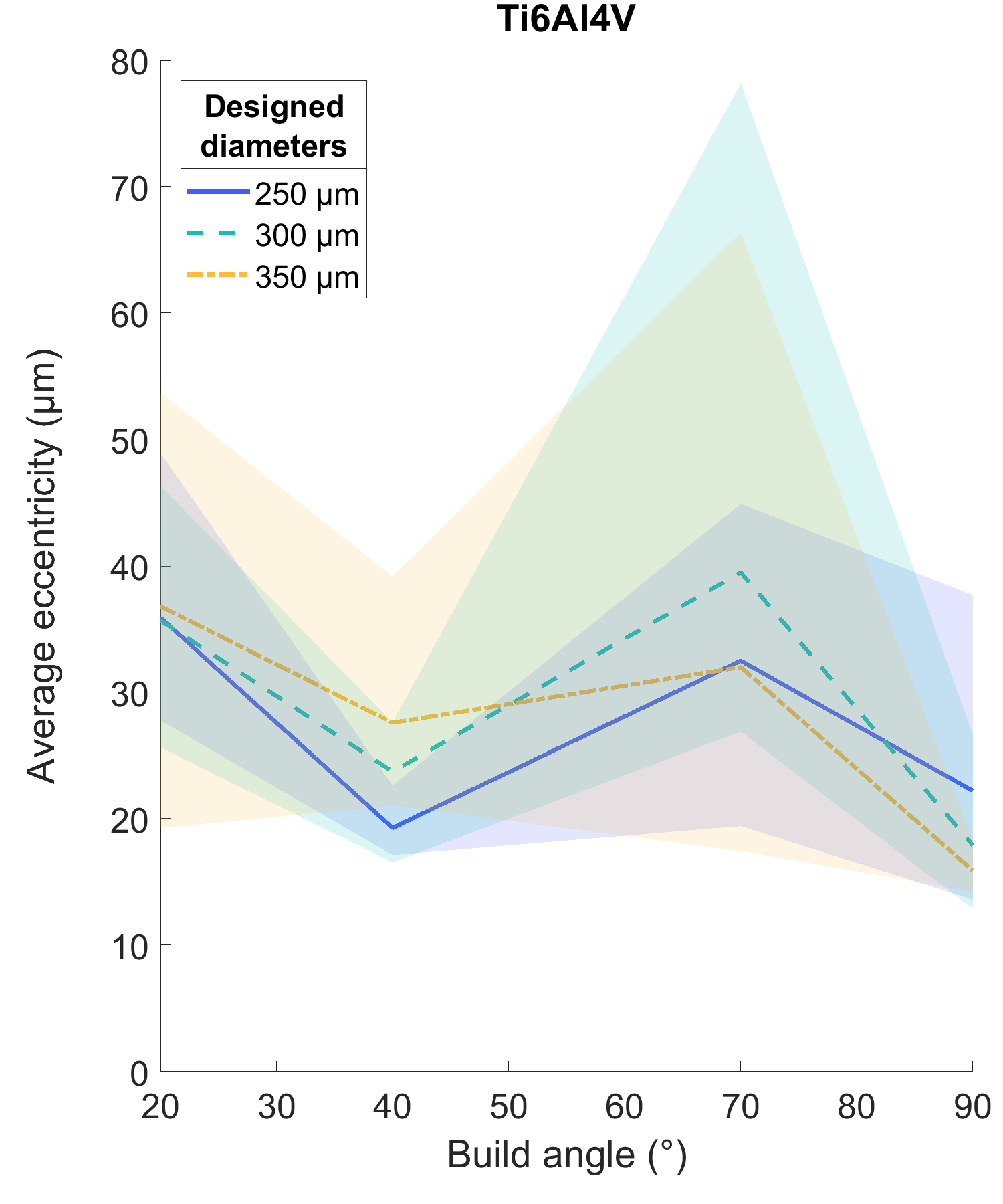 (b) |
| --- | --- |

Figure 15: Average eccentricity of the strut over build angle for (a) SS316L struts and (b) Ti6Al4V struts
